# Sizing up spotted lanternfly nymphs for instar determination and growth allometry

**DOI:** 10.1371/journal.pone.0265707

**Published:** 2023-02-02

**Authors:** Theodore Bien, Benjamin H. Alexander, Eva White, S. Tonia Hsieh, Suzanne Amador Kane

**Affiliations:** 1 Physics and Astronomy Department, Haverford College, Haverford, Pennsylvania, United States of America; 2 Department of Biology, Temple University, Philadelphia, Pennsylvania, United States of America; USDA Forest Service Southern Research Station, UNITED STATES

## Abstract

A major ongoing research effort seeks to understand the behavior, ecology and control of the spotted lanternfly (SLF) (*Lycorma delicatula*), a highly invasive pest in the U.S. and South Korea. These insects undergo four nymphal stages (instars) before reaching adulthood, and appear to shift host plant preferences, feeding, dispersal and survival patterns, anti-predator behaviors, and response to traps and chemical controls with each stage. However, categorizing SLF life stage is challenging for the first three instars, which have the same coloration and shape. Here we present a dataset of body mass and length for SLF nymphs throughout two growing seasons and compare our results with previously-published ranges of instar body lengths. An analysis using two clustering methods revealed that 1^st^-3^rd^ instar body mass and length fell into distinct clusters consistently between years, supporting using these metrics to stage nymphs during a single growing season. The length ranges for 2^nd^-4th instars agreed between years in our study, but differed from those reported by earlier studies for diverse locations, indicating that it is important to obtain these metrics relevant to a study’s region for most accurate staging. We also used these data to explore the scaling of SLF instar bodies during growth. SLF nymph body mass scaled with body length varied between isometry (constant shape) and growing somewhat faster than predicted by isometry in the two years studied. Using previously published data, we also found that SLF nymph adhesive footpad area varies in direct proportion to weight, suggesting that footpad adhesion is independent of nymphal stage, while their tarsal claws display positive allometry and hence disproportionately increasing grasp (mechanical adhesion). By contrast, mouthpart dimensions are weakly correlated with body length, consistent with predictions that these features should reflect preferred host plant characteristics rather than body size. We recommend future studies use the body mass vs length growth curve as a fitness benchmark to study how SLF instar development depends on factors such as hatch date, host plant, temperature, and geographic location, to further understanding of life history patterns that help prevent further spread of this invasive insect.

## Introduction

The spotted lanternfly (SLF), *Lycorma delicatula* White (Hemiptera: Fulgoridae) is a planthopper native to south Asia that has become a highly invasive pest in the U.S. and South Korea. SLFs feed intensively on phloem from a wide variety of trees and other plants, stressing the hosts as well as promoting the growth of sooty mold [1]. Because SLFs threaten significant economic damage to agricultural crops, native trees, and landscape plants, a large ongoing research effort seeks to understand their development, physiology, behavior and ecology to inform methods for mitigation and control [2–4]. In this study, we discuss how clustering methods can be applied to measurements of the body mass and size of immature SLFs (nymphs) in order to improve the determination of SLF life stage and to study the scaling of previously published SLF footpart and mouthpart dimensions [5] with body size. We begin by explaining how these issues are relevant to a wide variety of topics in SLF research.

**Fig 1:**
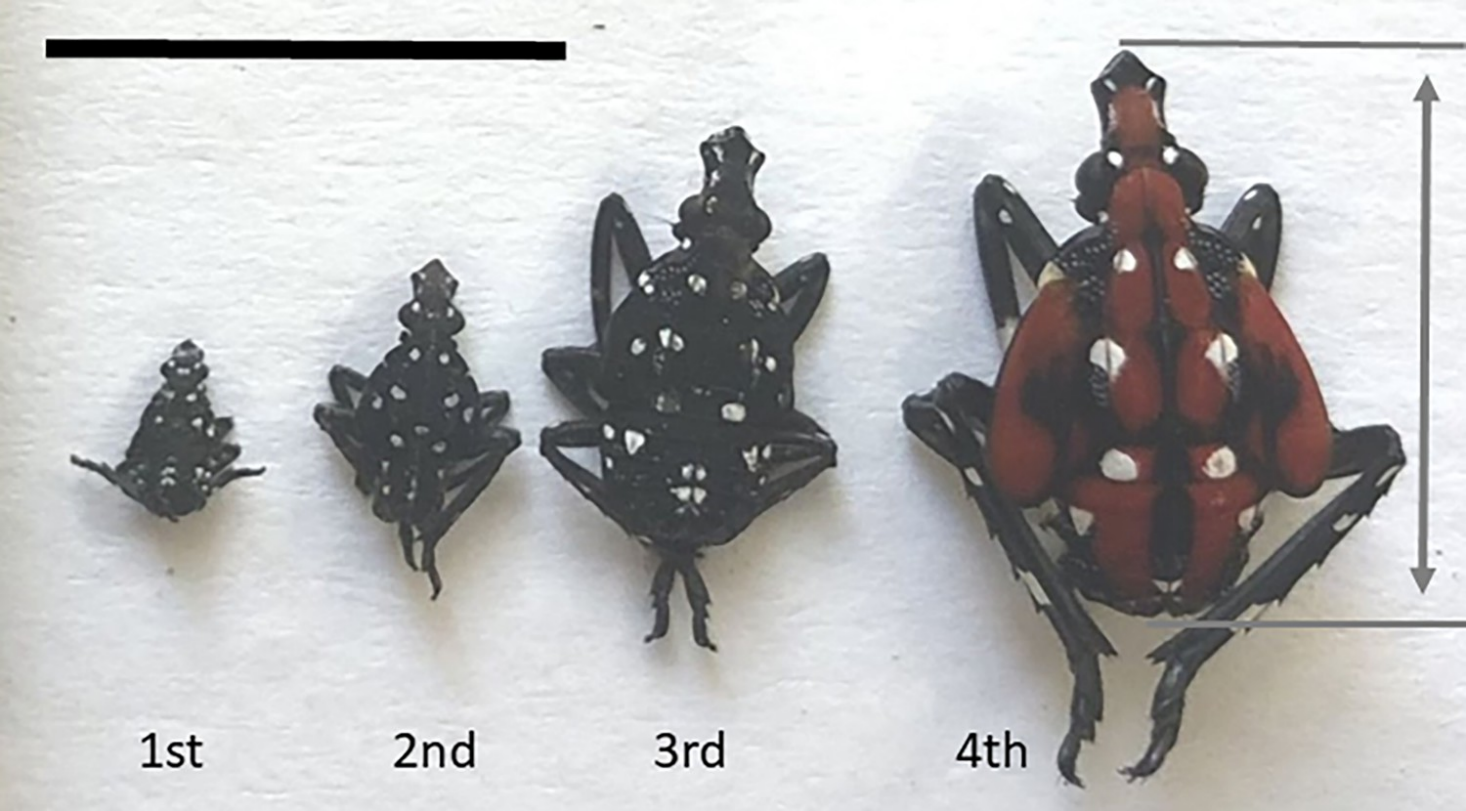
Photograph of 1st, 2nd, 3rd, and 4th instar spotted lanternfly nymphs. Double-headed arrow shows the definition of body length, L. (scale bar = 10 mm).

After emerging, SLFs develop through five life stages separated by molting: four nymphal instars and the much larger and winged adult stage. The 4th instars are readily identified by their distinctive red, black and white spotted coloration. However, the first through third instars have similar black-and-white-spotted coloration and overall body morphology (Fig 1) [6]. Many studies of SLF behavior, ecology, and phenology have relied on determination of the nymphal stage (instar determination) in order to track how life stage influences ecology and choice of host plants [1,2], dispersal patterns [3–5,7], locomotor behaviors such as climbing and jumping [8,9], phenology and activity [10], spectral preferences [6], attraction to chemicals [11], and effectiveness of various trapping methods [12]. Thus, instar determination methods for identifying the life stage of a given specimen collected in the field are useful and important in many contexts. Several previous studies have shown how a detailed microscopic examination can reveal foot, mouth part and antenna morphological changes during development [8,13,14], providing information of great utility for how these factors influence feeding, adhesion and locomotion throughout the insect’s life cycle. In practice, the life stages of the first three SLF instars have been estimated in many studies using overall body dimensions readily measured in the field, along with previously published size ranges for each instar.

In spite of this growing interest, only a few previous studies have reported measured data for the ranges of body lengths corresponding to each nymphal life stage for use in instar determination, and none have reported body mass. (Table 1, Fig 1) The earliest study reported only mean body lengths for each stage in China [15]. Park and colleagues [16] measured body lengths for 1st through 4th instars in South Korea, although it was not stated whether specimens used for measurements were raised in the laboratory with known life stage or collected from the wild and the instar stage estimated from size. Jang et al. [6] reported only body lengths for just 2nd instars captured in the field in South Korea. Dara et al. [17] reported the ranges of body lengths measured for 1st through 4th instar nymphs collected in Pennsylvania. None of these previous studies provided statistical data to guide the classification of new datasets for instar determination. Furthermore, studies have shown that the size ranges for each instar can depend on factors such as date of emergence, diet, host plants, temperatures, and environment (e.g., laboratory vs field-raised) [18]. Indeed, prior research has indicated that SLF nymphs develop and survive differently when reared with different diets in the field [19,20], at different temperatures [21], and artificial conditions (i.e., enclosures or laboratory conditions) [1,16], but these studies did not consider how these factors affected instar morphometrics.

**Table 1:** Body length (mm) of spotted lanternfly nymphs from this study and earlier work. N = number of specimens (not reported in [15]).

**Body length (mm)**	**Zhou, 1992** [15] **mean**	4	7	10	13
	**Park et al., 2009** [16] **mean ± SE**	3.9 ± 0.2 N = 43	5.7 ± 0.7 N = 62	8.9 ± 0.4 N = 23	11.6 ± 2.3 N = 37
	**Jang et al., 2013** [6] **mean ± SD**	Not measured	6.0 ± 0.5 N = 17	8.6 ± 0.5 N not given	11.3 ± 0.7 N not given
	**Dara et al., 2015** [17] **[range]**	[3.6, 4.4] N = 12	[5.1, 6.4] N = 10	[6.9, 9.4] N = 12	[10.9, 14.8] N = 10
	**This study 2021** **mean ± SD**	4.24 ± 0.24 N = 54	6.67 ± 0.48 N = 30	9.30 ± 0.72 N = 61	11.74 ± 0.75 N = 49
	**This study 2022** **mean ± SD**	4.32 ± 0.35 N = 49	6.73 ± 0.38 N = 88	9.50 ± 0.54 N = 49	12.28 ± 0.60 N = 40
	**Life stage**	**1**^**st**^ **instar**	**2**^**nd**^ **instar**	**3**^**rd**^ **instar**	**4**^**th**^ **instar**

For many insects and other arthropods, instar determination (i.e., identifying the life stage of nymphs collected in the field) relies on the observation that major changes in body dimensions occur primarily when the exoskeleton is shed upon molting [18]. Ideally, this involves directly measuring the frequency distribution of one or more metrics of exoskeleton size for each instar using laboratory-reared specimens with known molting status (e.g., based on molted head capsule dimensions) [22]. However, instar determination should be possible without knowledge of molting status if the number of developmental stages is known in advance, the morphometric data are uniformly sampled across all life stages, and the frequency distribution of these data is partitioned into distinct clusters [23]. The last approach is especially useful for SLFs, which have proven challenging to raise in the laboratory so that life stage can be directly monitored [20,21], and which we have observed to have flaccid cast exoskeletons that do not provide useful sizing information after molting.

In this study, we report measurements of mass and body length for spotted lanternfly nymphs along with clustering results for these specimens. By comparing these results with those from four previous studies, we explore the variation in SLF nymph size distributions reported thus far. A growth (ontogenetic) allometric analysis was also performed to identify possible adaptations for feeding morphology and biomechanics. Body mass has been found to scale as a power law of body length (i.e., M = L^c^) for a wide range of insect and other arthropod taxa with scaling exponents c that vary from < 1 to 3 [24], where c = 3 corresponds to isometric growth (geometrical similarity; maintaining a constant shape), c > 3 to positive allometry (i.e., growing faster than predicted by isometry) and c < 3 to negative allometric growth (i.e., growing more slowly than predicted by isometry). We used our data to determine the ontogenetic scaling regime of SLF nymph body mass, and use previously-published morphometric data to determine how the dimensions of foot and mouth body parts scale with overall body size. We interpret these results in relation to SLF behavior and ecology, and suggest ways these methods can be applied in future work.

## Methods

### Insect collection and morphometrics

Healthy, intact SLF nymphs were collected in the field from *Ailanthus altissima* trees and wild grape vines (*Vitis spp*.) in southeastern Pennsylvania (40°00’30.2"N 75°18’22.0"W) from May through July, 2021 and 2022, corresponding to 1st instar emergence until it was difficult to find 4^th^ instars (note this was a different date in the two years). In both years, we measured all specimens collected from the field site using an insect net to avoid sampling bias. Details on the collection timeline and number of specimens collected, which corresponded to multiple samples per instar (2–4 weeks/stage in 2021; 4–5 weeks/stage in 2022) are given in S1 Appendix. A total of N = 194 (2021) and N = 226 (2022) specimens were collected across all nymphal life stages (Table 1). Because SLF are identified as an invasive species in Pennsylvania, all specimens were euthanized by freezing [25].

Specimens were gathered from the insect net using scoop-shaped forceps to avoid damage and placed immediately in plastic containers that contained a damp paper towel (to maintain humidity) and freshly-picked *A*. *altissima* leaves still on branches (as a food source). They were then placed in air-tight plastic containers, frozen, and stored at a constant -15 deg C within two hours of collection. Morphometric data were measured post-mortem after thawing for 15 min at 23.0 ±1.2 deg C and relative humidity 56 ± 14% to preserve tissue hydration and morphology (model DVTH DataView logger, Supco). Specimens were handled using featherweight entomology forceps and were not placed under any stress at any point during storage or measurement to avoid distortion of their bodies. Body mass, M, was measured using an analytical balance (Explorer, Ohaus, Parsippany, NJ US) to ± 0.4 mg accuracy. Body length, L, was defined as distance between the anterior end of the head to the posterior end of the abdomen. We measured body length using ImageJ [26] to ± 0.05 mm from digital micrograph images of specimens lying flat on their dorsal or ventral surfaces with a scale bar in the same plane; using a digital caliper resulted in identical measurements within instrumental uncertainty. Micrographs taken from a variety of perspectives indicated that the effect of specimen orientation was ≤ 4% of body length and hence less than or equal to measurement uncertainty. We measured the body length of dead specimens because live nymphs stand with their bodies tilted relative to the surface by 20 to 33 deg (measured from side view photographs taken of N = 18 live 3^rd^ and 4^th^ SLF instars). When live specimens are viewed from above, this body tilt foreshortens the apparent body length and other dimensions by a factor ranging from 0.84 to 0.94, necessitating the use of close-up images recorded at more than one angle. We did not consider sexual dimorphism because an earlier study [6] found only a small (< 4%) difference in median body lengths between sexes for 4^th^ instar nymphs and reported difficulty in determining the sex of earlier instars.

### Comparison with other studies

We performed Google Scholar searches using the keywords spotted lanternfly and *Lycorma delicatula*, yielding over 600 references. Approximately 100 papers that directly studied SLFs were used to perform repeated forward and reverse citation searches to find morphometric data for spotted lanternfly nymphs. This resulted in the identification of four papers with additional values of body length [6,15–17]. We also found one study that reported adult SLF body length and mass, which agreed with our own observations [27]. One study reported morphometric data for footpart and mouthpart dimensions [14]; here we consider their values for the tarsal claw tip-to-tip distance (their TCT), the area, A_adh_, of the arolium (adhesive pad) estimated from their arolium morphometric data, as well as the lengths of the labium, L_L_, and stylet, L_S_, which is used to pierce plant surfaces for feeding. (See S2 Appendix for more information about footpart measures.)

### Data analysis and statistics

Data analysis was performed using MATLAB version R2021a with the curve fitting and statistics and machine learning toolboxes (Mathworks, Natick MA USA); MATLAB functions are referred to using italicized names. Results are reported as mean [95% CI] unless indicated otherwise. All data and code required to reproduce all results and figures discussed here are included in S1 Dataset.

All 4th instars were identified by their red, black and white coloring. Length and mass data for all specimens with black and white coloration consistent with 1st through 3rd instars were standardized before clustering by converting them into z-scores (i.e., zero mean and standard deviation = 1). For the first clustering method, the standardized data were fit to a three component Gaussian Mixture Model using *fitgmdist* (covariance type = full, shared covariance = false), then sorted into three components (clusters) using *cluster* in MATLAB to reflect the known number of instar stages in the dataset. We also partitioned only the length data for the first, second and third instars into three clusters using the Gaussian Mixture Model and *kmeans* for k-means clustering.

We next analyzed the mean lengths for each estimated instar to determine whether they follow Dyar’s Rule, the observation that instar body dimensions increase in size by a constant growth ratio between successive instars [28] This implies that log L_j_ = j × log G + log L_0_, where j = instar number, L _j_ = mean body length of the jth instar, and G = growth ratio = L_j+1_ / L _j_ [18]. We used simple ordinary linear regression (MATLAB *fitlm*) to fit body length data from this study and previous work vs estimated instar number; we also computed as goodness-of-fit measures the F-statistic and p-value for significance testing (alpha = 0.05; null hypothesis no dependence on the independent variable), and R-squared. We used MATLAB *confint* to find 95% CI of all fit parameters. To fit body length data from previous studies, we used either means or middle of the quoted range, depending on which statistics were provided. (Table 1)

To determine the power law dependence of body mass on body length (i.e., M = a L^c^), we first log-transformed the M and L data, and then used simple ordinary linear regression to fit to log M = a + c log L, as described above. The fitted slope (the scaling exponent, c) was used to determine whether these data were consistent with the null hypothesis of isometric scaling (i.e., c = 3), or instead with positive or negative allometry. (See full results in S4 Appendix).

We performed the same analysis for tarsal claw and mouthpart (stylet and labium) dimensions from a previous study [14] vs body length to test for agreement with power law scaling with body length, and isometric scaling(c = 1) in particular. Because scaling law fits to both mouthpart lengths vs body length did not include any adult data within the fit confidence intervals, we also performed fits to only the data for nymphs.

Previous research has found that the adhesive pad area, A_adh_, scales linearly with body mass for organisms over a wide range of taxa and body sizes [29]. For comparison, isometric scaling predicts that A_adh_ ∞ L^2^ ∞ M^2/3^. We therefore tested whether either of these relationships hold for SLFs using published data for their arolium dimensions [14] to estimate the arolium area (S2 Appendix); we then performed scaling law fits to these data vs body mass using the methods described above. Because the arolium does not increase monotonically in size (i.e., it is smaller on average for adults than for 4th instars) we fitted only the values for nymphs.

## Results

### Instar determination

**Fig 2:**
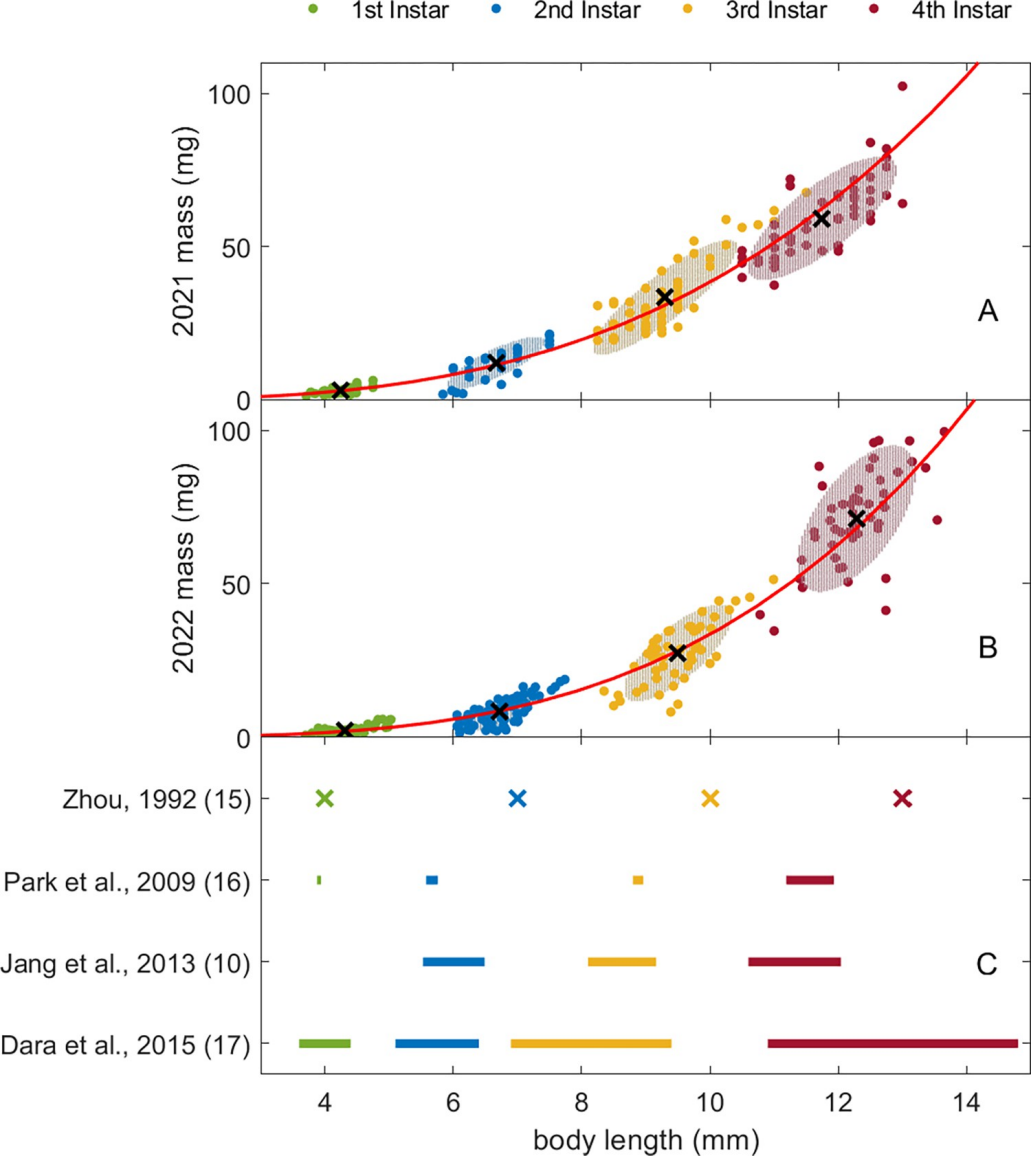
Clustering of spotted lanternfly nymph mass vs length data. Body mass vs length (filled circles) for (A) 2021 and (B) 2022 data; cluster centroids are shown as black x markers and red lines indicate scaling law fits to all data. Shaded ellipses show the 95% CI for each cluster based on the Mahalanobis distance; note that the shaded ellipses for some clusters are covered by datapoints. (C) Symbols and horizontal lines show the means and ranges of lengths, respectively, for each instar reported in previous studies (Table 1).

**Fig 3:**
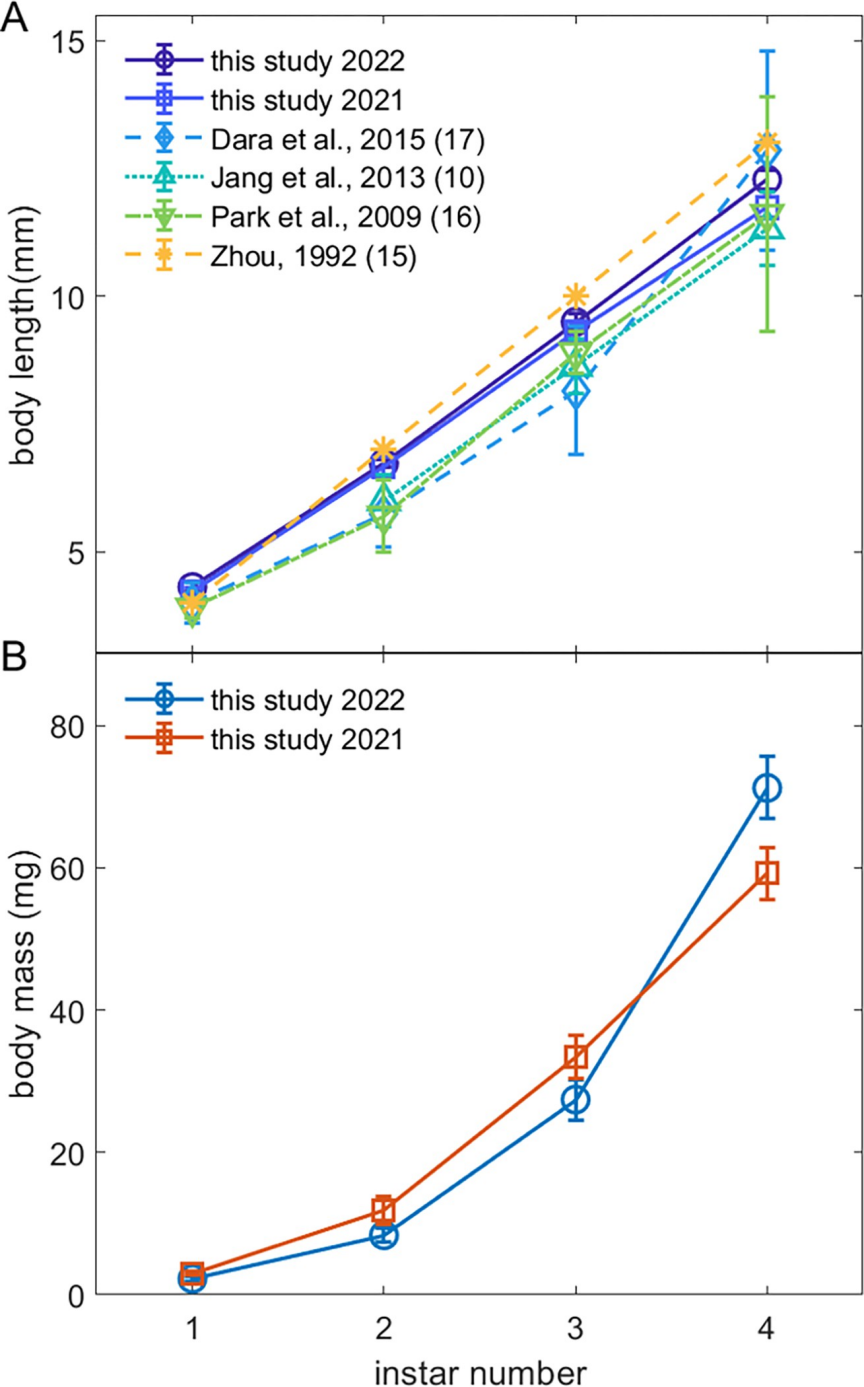
Comparison of body length and mass for each spotted lanternfly nymph stage from this study and previous research. (A) Spotted lanternfly nymph body length vs instar from this study and previous work (Table 1). (B) Spotted lanternfly nymph body mass vs instar from this study. Error bars are 95% CI for this study and the measures of variance given in Table 1. (Lines between datapoints show overall trends).

The results of our morphometric measurements are shown in Fig 2 along with clustering data using the GMM model for mass vs body length. (See full results in S3 Appendix.) The data were sorted into identical clusters using GMM clustering for mass vs length and using k-means on lengths only. The cluster centroids provide estimates for the mean body length and mass for each life stage, which we compare with earlier studies in Fig 3. As can be seen in Fig 3A and Table 1, the body lengths for each instar agreed closely for the two years studied here, but not with values from earlier studies. The body masses were lower for early instars for the 2022 data than for those in the 2021 data, but greater for 4^th^ instars.

**Fig 4:**
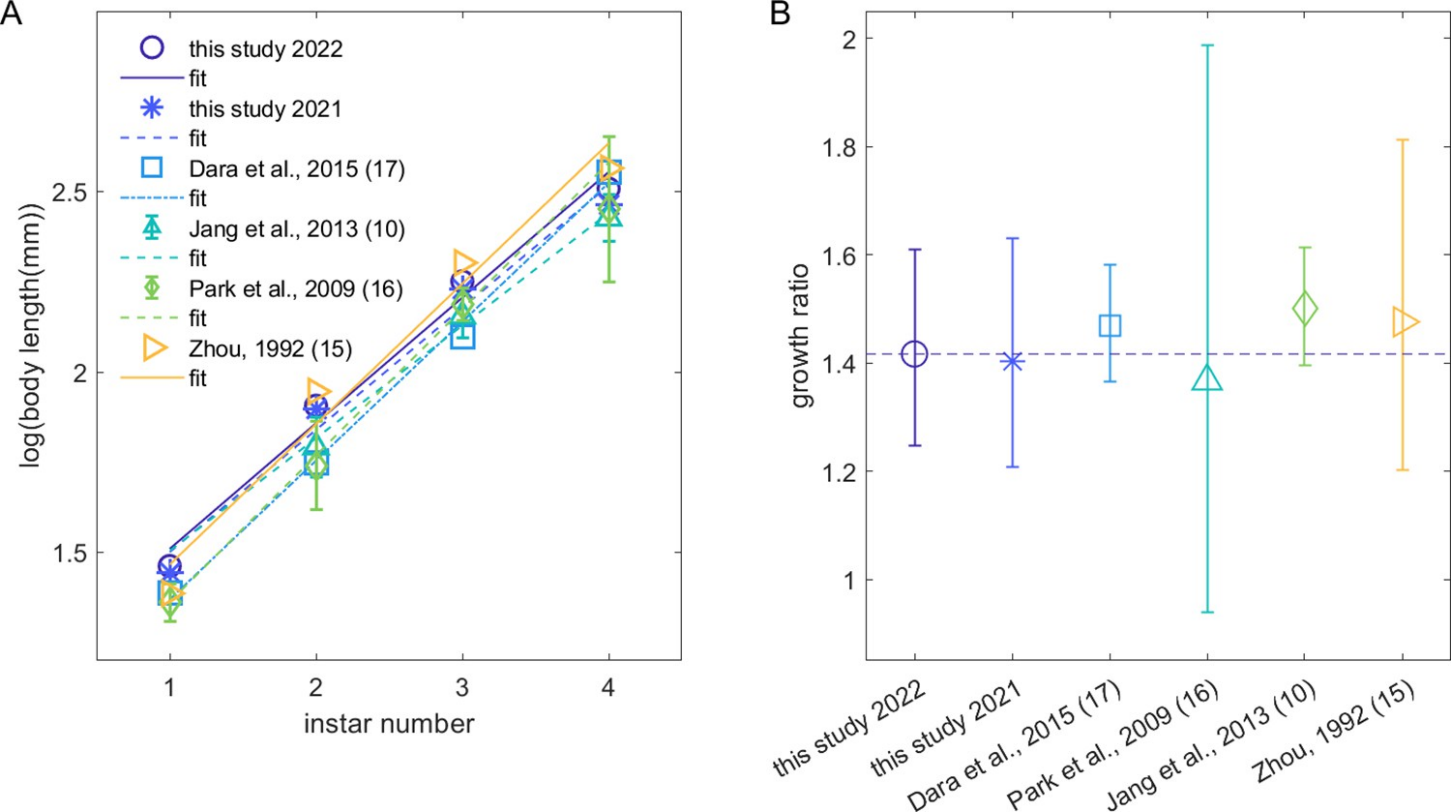
Results from Dyar’s Rule analysis of data from this study and previous research. (A) Plots of spotted lanternfly nymph log(body length) plotted vs estimated instar number as a test of Dyar’s rule. Markers show data from this study and three previous papers, while lines indicate linear fits. (B) Growth ratios, G, from the fits in (A); dashed line indicates the mean value for 2022 from this study. All error bars are 95% CI.

Fig 4A show the results from testing Dyar’s Rule for SLF instar body length for this study and four previous studies. (See S4 Appendix for full results.) For every study except [6], body length was significantly related to instar number and the linear fit explained over 97% of total variance in the data in agreement with Dyar’s Rule. The fitted growth ratio for our 2022 data, G = 1.42 [1.25, 1.61], was consistent with that from 2021 and earlier studies (p > 0.95) (Fig 4B).

### Allometry

**Fig 5:**
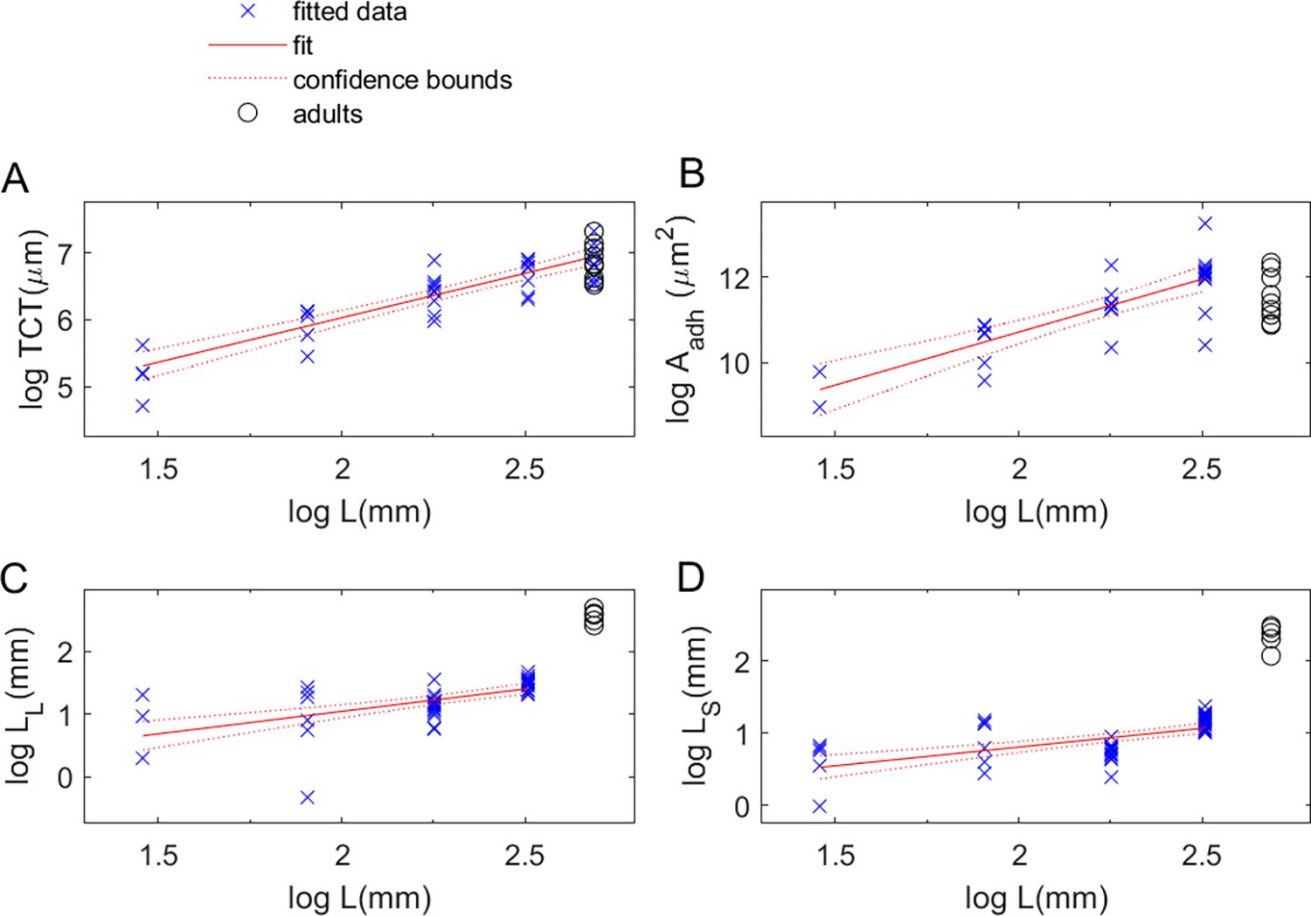
Plots and fits showing how spotted lanternfly foot and mouthpart data relate to body size measures. A) Plots of data and fit results for the dependence on log body length for (A) the log tarsal claw tip-to-tip width, TCT, (B) arolium area, A_adh_, (C) labium length, L_L_, and (D) stylet length, L_S_. Footpart and mouthpart morphometric data are from [14], while nymph body length and mass are from this study and adult mass and length from [27]. (Fit lines and 95% confidence intervals from ordinary linear regression fits to power laws, as described in the main text).

The SLF nymph body mass vs length data followed power law scaling with significantly different scaling exponents for the two years studied: 3.01 [2.94, 3.09] for 2021 data and 3.45 [3.34, 3.56] for 2022 (Fig 2); the results for 2021 were thus consistent with isometric scaling (c = 3) while those for 2022 indicated somewhat positive allometry (c > 3 by 13%). (See S4 Appendix for full results for all scaling law fits.) Given the close agreement between instar length distributions in 2021 and 2022, we only used 2022 data in the analysis of foot and mouthpart data from [14]. Fit results revealed that power law models accounted for a high percent of total variance for the footpart dimensions considered (77% and 62% for TCT and A_adh_, respectively). This also showed that SLF tarsal claw tip-to-tip distance [14] scales with body length over all SLF life stages with c = 1.33 [1.09, 1.56], indicating that this measure of the tarsal claws’ grasp displays positive allometry (i.e, c > 1). (Fig 5A) SLF nymph arolium area depended on L with scaling exponent c_L_ = 2.49 [1.75, 3.23]; this corresponded to A_adh_ ∞ M ^0.89 ± 0.27^ (2021) and M ^0.73 ± 0.22^ (2022), in agreement with the dependence found for other taxa (S1 Appendix, [29] but not with isometry (A_adh_ ∞ M^2/3^). (Fig 5B) By contrast, the fits shows that SLF nymph mouthpart dimensions (labium length, L_L_, and stylet length, L_S_) were only weakly correlated with body length (S4 Appendix, Fig 5B and 5C); i.e., power law scaling explains only 36% of the total variance in these data.

## Discussion

The results of this study lead to several conclusions. First, the distribution of measured data for body mass and length for 1^st^ to 3^rd^ SLF instars were consistent with three non-overlapping clusters of data in the approximate size ranges expected for these life stages from previous studies. We consequently used two methods to assign these data to three clusters: 1) GMM on both mass and length data; 2) k-means clustering applied to length-only data. Both methods resulted in identical cluster assignments. We also found consistent values for the growth ratio between instars using Dyar’s Rule for all studies considered here.

This indicates that easy-to-perform specimen body length measurements and k-means clustering should be sufficient for instar determination. (In contrast to the distinct ranges found for 1^st^ to 3^rd^ instars, the measured body length and mass data ranges for 3rd and 4th instars overlapped in both years of our study. Due to the red coloration of 4th instar nymphs, however, this overlap in ranges does not cause a logistical challenge for properly categorizing these life stages.) The maximum range of variation in the body length of 1^st^ through 3^rd^ instars were equal to 13–16% of the mean, confirming our expectation that the <4% variation reported between sexes [6] would be small compared to the overall variation in body length at each nymphal life stage.

Practical applications of these findings include the indication that clustering methods used with an appropriate dataset of spotted lanternfly body lengths should facilitate determining the likely developmental stage of individual specimens in future studies. The clustering code provided could be used by other researchers to estimate the life stage of new specimens by performing clustering on their own measurements, or our data (assuming environments and other conditions similar to those in our study).

Second, we consider the variability in these measures of body size. Whereas the body length data were consistent for all nymphal life stages between the two years of our study, there was considerable variation between our results and amongst the ranges found by other studies for 2^nd^-4^th^ instars. We also found small but significant differences (-17% to +9%) between the 2021 and 2022 data for mean body masses at each life stage. This diversity of reported values suggests that the range of each instar’s body size may indeed vary among different seasons and environments. Additional data collected with uniform sampling and measurement methods could help resolve whether time of first emergence, temperature, or other environmental factors influence the mass and length at different life stages. For example, these metrics could be incorporated into studies of insect-plant interactions to elucidate the effect of diet on SLF growth.

Third, these data also provide insights into the ontogenetic scaling of spotted lanternfly nymph body metrics. Body mass scaled with body length for SLF nymphs with different exponents consistent with an approximately constant overall geometric shape (isometric scaling, 2021) or slightly positive allometry (2022). This approach can be used with existing data to provide insight into the biology of these insects. For example, Kim et al. [30] hypothesized that earlier SLF instars should be more easily dislodged by wind than later nymphs due to their smaller arolia, an idea with implications for how dispersal and control should depend on life stage. However, SLF nymph arolium area (the morphometric measure relevant for adhesive strength) from [14] was found to scale with extreme positive allometry with body length and mass, in agreement with the scaling relationship found across taxa over 7 orders of magnitude of body weight [29]. In combination with the finding of constant (i.e., size-independent) maximum adhesive stress between the arolium and surface for other insect adhesive pads (e.g., the pulvilli of *Coreus marginatus* [30] and the arolia of stick insects in [31]) across all life stages, this implies that SLF instars could have similar adhesive capabilities across life stages. The reported monotonic increase in SLF falling-climbing cycle period with advancing date of the year [8] could be due to factors other than arolium development, such as faster than isometric growth of tarsal claw grasp along with the detailed morphometric changes reported in [14] (increased wrinkling of the arolia surface and a larger terminal sticky lip in adult SLFs relative to nymphs). These findings for adhesion are of especial interest because of the crucial role transportation plays in the dispersal of SLFs, which are known to travel long distances by clinging to vehicles and shipping containers [32].

By contrast, the analysis showed that the variation in the stylet and labium lengths was only weakly correlated with body length for SLF nymphs [14]. This is consistent with the expectation that stylet length is correlated with preferred host plant tissue characteristics [33], as opposed to insect size, given reports from the literature indicate that SLF nymphs only feed on herbaceous and non-woody parts of plants (e.g., shoots, stems and leaves) while adults are able to feed on bark-covered trunks [8,10,16,17,34].

## Conclusion

We propose that body mass vs length curves could play a role similar to that of clinical growth charts, filling the current gap in metrics of SLF development. These measures could serve as a much-needed fitness benchmark [35] for interpreting data from field studies and laboratory experiments to assess the impact of factors such as date of first emergence, molt schedule, temperature, diet, and geographic location. Furthermore, the successful fits to Dyar’s rule provide a measure of the growth ratio between successive instars, which might serve as an additional metric for comparing populations grown under different circumstances. Another potential use of these methods involves estimating the life stage of isolated SLF nymphs found in new locations as these insects expand their range. This information can play a valuable role in determining the stage of infestation, informing control efforts as well as providing data useful for tracking and modeling their spread. We therefore suggest that morphometrics of SLF nymphs be incorporated into ongoing studies when possible so as to provide a wide range of data for such applications going forward.

## Supporting information

S1 Dataset All data and code required to reproduce the figures and results in the text. (ZIP)

S1 Appendix Sampling timeline for spotted lanternfly nymph body length and mass. (PDF)

S2 Appendix Spotted lanternfly arolium area. (PDF)

S3 Appendix Clustering results. (PDF)

S4 Appendix Full fitting & data analysis results. (PDF)
