# Supplementary material for: Sizing up spotted lanternfly nymphs for instar determination and growth allometry
Source: PLoS One. 2023 Feb 2;18(2):e0265707. doi: 10.1371/journal.pone.0265707 (PMC9894384; doi:10.1371/journal.pone.0265707)
Supplement: S1 Dataset — (ZIP) [file pone.0265707.s001.zip › SizingUpSLFs_metadata.docx]

Sizing up spotted lanternfly nymphs for instar determination and allometry

Theodore Bien^1^, Benjamin H. Alexander^1^, Eva White^1^, S. Tonia Hsieh^2^, Suzanne Amador Kane^1^

^1^ Physics and Astronomy Department, Haverford College, Haverford, Pennsylvania, United States of America

^2^ Department of Biology, Temple University, Philadelphia, United States of America

Metadata for S1 Dataset (code & data available on figshare)

Note that the MATLAB code assumes the data files are stored in the same folders as the code itself.

1. SLFBothYearDistributions_v2.m Creates Fig. 2 plots.

Data: SLFNymphMassLengthData.xlsx

1. plot_all_LandMdata.m: Creates Fig 3 plots.

Data: SLFNymphMassLengthData.xlsx

1. DyarsRulePlots.m: Creates Fig. 4 plots

Data: SLFNymphMassLengthData.xlsx

1. FootMouthMorphAllometry.m: Creates Fig. 5: plots other SLF body measures vs body length and mass.

Data: SLFFootMouthPartData.xlsx

1. sampling_timeline_plots_v2.m Creates S1 Fig.

Data: Sampling Timeline.xlsx

1. SLFClustering.m Performs GMM clustering and fitting to an allometric scaling law for the data for spotted lanternfly (SLF) nymph body mass vs body length from this study, and the SLF nymphs body lengths reported in four earlier studies.

Data: SLFNymphMassLengthData.xlsx

Outputs: S4 & S5 Fig, Appendix 2
