# Supplementary material for: Sizing up spotted lanternfly nymphs for instar determination and growth allometry
Source: PLoS One. 2023 Feb 2;18(2):e0265707. doi: 10.1371/journal.pone.0265707 (PMC9894384; doi:10.1371/journal.pone.0265707)
Supplement: S1 Appendix — (PDF) [file pone.0265707.s002.pdf]

Sizing up spotted lanternfly nymphs for instar determination and growth allometry

Theodore Bien[1], Benjamin H. Alexander[1], Eva White[1], S. Tonia Hsieh[2], Suzanne Amador Kane[1]

[1] Physics and Astronomy Department, Haverford College, Haverford, Pennsylvania, United States of America

[2] Department of Biology, Temple University, Philadelphia, United States of America

S1 Appendix. Sampling timeline for spotted lanternfly nymph body length and mass

S1 Table. Number of weeks over which each nymphal life stage was sampled.

	**Number of weeks collected**	
**Life stage**	**2021**	**2022**
1st instar	2	5
2nd instar	4	5
3rd instar	3	5
4th instar	3	4

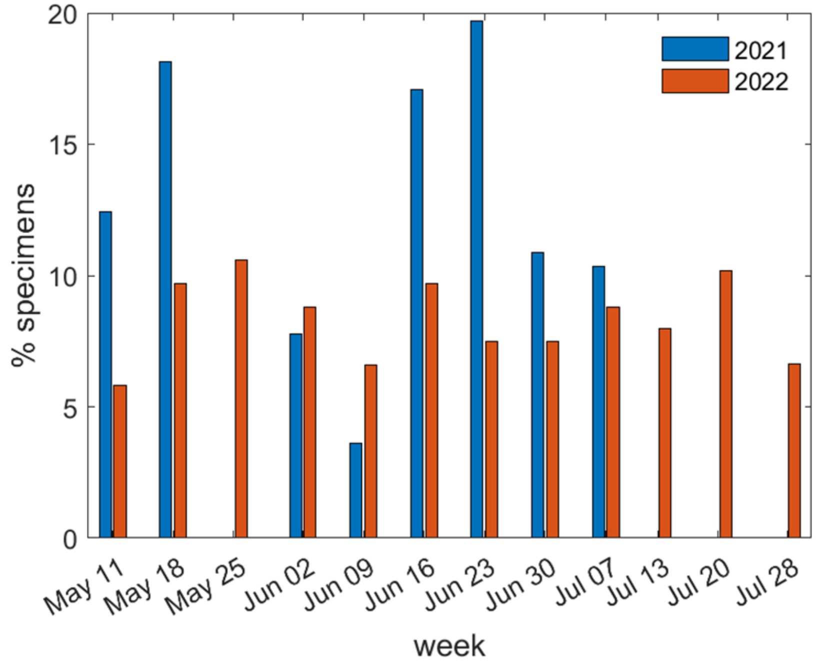

S1 Fig. Sampling timeline for summer 2021 & 2022
