# Supplementary material for: Sizing up spotted lanternfly nymphs for instar determination and growth allometry
Source: PLoS One. 2023 Feb 2;18(2):e0265707. doi: 10.1371/journal.pone.0265707 (PMC9894384; doi:10.1371/journal.pone.0265707)
Supplement: S2 Appendix — (PDF) [file pone.0265707.s003.pdf]

Sizing up spotted lanternfly nymphs for instar determination and growth allometry

Theodore Bien[1], Benjamin H. Alexander[1], Eva White[1], S. Tonia Hsieh[2], Suzanne Amador Kane[1]

[1] Physics and Astronomy Department, Haverford College, Haverford, Pennsylvania, United States of America

[2] Department of Biology, Temple University, Philadelphia, United States of America

S2 Appendix. Spotted lanternfly arolium area

We used the data from Table 5 in (1) for spotted lanternfly arolium (foot adhesive pad) dimensions using the approximately triangular geometry defined in Fig 1 in ref. (1). This gives an estimated arolium area, A_{adh}:

$$A_{adh} = \frac{1}{2} \times A1 \times A2\, sin\left(90° - {}^{\theta}/_{2}\right),$$

where the relevant arolium dimensions are defined in S2 Fig and S2 Table below.

S2 Table. Definitions of labels for arolium dimensions.

	Label in Fig 1 ref. (1)	Label in Table 5 ref. (1)
maximum anterior width of the arolium	A1	AAW
length of the lateral margin of the arolium	A2	ASL
angle between the lateral margins of the arolium from the dorsal view	θ	AAG

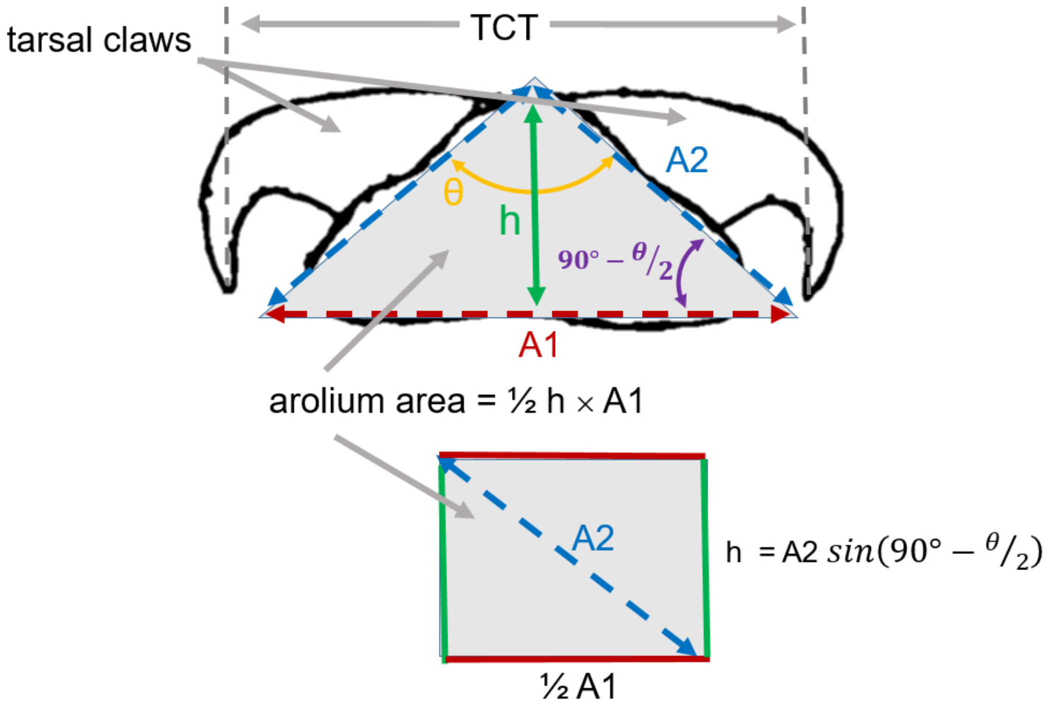

S2 Fig. Approximately triangular geometry used to define the arolium's dimensions. TCT = tarsal claw tip separation (adapted from Fig 1 in ref. (1)).

References

1. Avanesyan A, Maugel TK, Lamp WO. External morphology and developmental changes of tarsal tips and mouthparts of the invasive spotted lanternfly, Lycorma delicatula (Hemiptera: Fulgoridae). PLoS One [Internet]. 2019 Dec 26 [cited 2020 Jun 16];14(12). Available from: https://www.ncbi.nlm.nih.gov/pmc/articles/PMC6932783/
2. Frantsevich L, Ji A, Dai Z, Wang J, Frantsevich L, Gorb SN. Adhesive properties of the arolium of a lantern-fly, Lycorma delicatula (Auchenorrhyncha, Fulgoridae). Journal of Insect Physiology. 2008 May 1;54(5):818–27.
