# Supplementary material for: Sizing up spotted lanternfly nymphs for instar determination and growth allometry
Source: PLoS One. 2023 Feb 2;18(2):e0265707. doi: 10.1371/journal.pone.0265707 (PMC9894384; doi:10.1371/journal.pone.0265707)
Supplement: S3 Appendix — (PDF) [file pone.0265707.s004.pdf]

Sizing up spotted lanternfly nymphs for instar determination and growth allometry

Theodore Bien[1], Benjamin H. Alexander[1], Eva White[1], S. Tonia Hsieh[2], Suzanne Amador Kane[1]

[1] Physics and Astronomy Department, Haverford College, Haverford, Pennsylvania, United States of America

[2] Department of Biology, Temple University, Philadelphia, United States of America

S3 Appendix. Clustering results

Table S3. Morphometric data for spotted lanternfly nymph mass and body length. The 4th instars were identified by coloration, while 1st, 2nd and 3rd instars were classified using fits of the mass vs length to a 3 component Gaussian mixture model (see Methods in main text for details). (N = number specimens. All values are given as mean ± SD.)

**Life stage**	**N 2022**	**Body length (mm) 2022**	**Body mass (mg) 2022**	**N 2021**	**Body length (mm) 2021**	**Body mass (mg) 2021**
1st instar	49	4.32 ± 0.35	2.2 ± 1.4	54	4.24 ± 0.24	3.0 ± 1.2
2nd instar	88	6.73 ± 0.38	8.4 ± 4.2	30	6.67 ± 0.48	12.0 ± 5.5
3rd instar	49	9.50 ± 0.54	27.4 ± 10.2	61	9.30 ± 0.72	33.4 ± 12.0
4th instar	40	12.28 ± .60	71.2 ± 15.4	49	11.74 ± .75	59.2 ± 13.0
