# Supplementary material for: Sizing up spotted lanternfly nymphs for instar determination and growth allometry
Source: PLoS One. 2023 Feb 2;18(2):e0265707. doi: 10.1371/journal.pone.0265707 (PMC9894384; doi:10.1371/journal.pone.0265707)
Supplement: S4 Appendix — (PDF) [file pone.0265707.s005.pdf]

# Sizing up spotted lanternfly nymphs for instar determination and growth allometry

Theodore Bien[1], Benjamin H. Alexander[1], Eva White[1], S. Tonia Hsieh[2], Suzanne Amador Kane[1]

[1] Physics and Astronomy Department, Haverford College, Haverford, Pennsylvania, United States of America

[2] Department of Biology, Temple University, Philadelphia, United States of America

## S4 Appendix. Full fitting & data analysis results

**S4 Table. Results from fits to Dyar's Rule for data from this study and previously-published work.** Fit to Dyar's Rule: $\log L_j = j \times \log G + \log L_0$, where j = instar number, $L_j$ = mean body length of the jth instar, and G = growth ratio = $L_{j+1} / L_j$ (dof = fit degrees of freedom. Note that when $\log L_0$ is not significantly different from 0, this simply means that $L_0 = 1$ and does not indicate a poor fit to the allometric scaling law.)

| Data | log L | t | p | G | t | p | R-squared | F | P | dof |
|---|---|---|---|---|---|---|---|---|---|---|
| this study 2022 | 1.16 ± 0.08 [0.81, 1.51] | 14 | 0.005 | 1.42 ± 0.04 [1.25, 1.61] | 12 | 0.007 | 0.986 | 138 | 0.007 | 2 |
| this study 2021 | 1.16 ± 0.10 [0.75, 1.57] | 12 | 0.007 | 1.40 ± 0.05 [1.21, 1.63] | 9.7 | 0.01 | 0.979 | 95 | 0.01 | 2 |
| Dara et al.. 2015 (17) | 0.98 ± 0.05 [0.78, 1.18] | 21 | 0.002 | 1.47 ± 0.02 [1.37, 1.58] | 23 | 0.002 | 0.996 | 518 | 0.002 | 2 |
| Jang et al., 2013 (10) | 1.19 ± 0.10 [-0.02, 2.41] | 12 | 0.05 | 1.37 ± 0.04 [0.94, 1.99] | 11 | 0.06 | 0.991 | 112 | 0.06 | 1 |
| Park et al., 2009 (16) | 0.96 ± 0.04 [0.78, 1.13] | 24 | 0.002 | 1.50 ± 0.03 [1.40, 1.61] | 24 | 0.002 | 0.997 | 583 | 0.002 | 2 |
| Zhou et al., 1992 (15) | 1.08 ± 0.13 [0.52, 1.64] | 8.3 | 0.014 | 1.478 ± 0.07 [1.20, 1.81] | 8.2 | 0.01 | 0.971 | 67 | 0.01 | 2 |

**S5 Table. Full results from allometric fits to y = a + c log x.** Log-transformed values for the spotted lanternfly body mass = M (mg), tarsal claw tip distance = TCT (μm), nymph labium length = $L_L$ (mm) and stylet length = $L_S$ (mm) from (14) were fitted vs log-transformed body length = L (mm) from this study and (27). Log arolium area = $A_{adh}$, (micrometer$^2$) computed from data in (14) was fitted to log M from this study and (27). Fit parameters are intercept = a, and slope, c = scaling exponent. For M vs L fits, because some specimens has residuals that were outliers due to low mass (as determined using MATLAB *isoutlier* , < 3 median absolute deviation threshold), with disproportionately flat abdomens presumably due to hunger), we conducted fits with and without excluding outliers (5 for 2021 and 13 for 2022 data); scaling exponents for these fits differed by 1% so we report only results excluding outliers here.

| **Data** | **Intercept, a** | **t** | **p** | **scaling exponent, c** | **t** | **p** | **R-squared** | **F** | **P** | **dof** |
|---|---|---|---|---|---|---|---|---|---|---|
| M vs L (2022) | -4..42 ± 0.11 [-4.65, -4.20] | -39 | < 0.001 | 3.45 ± 0.07 [3.34, 3.56] | 48 | < 0.001 | 0.948 | 2323 | < 0.001 | 211 |
| M vs L (2021) | -3.29 ± 0.08 [ -3.44, -3.13] | -43 | < 0.001 | 3.01 ± 0.04 [2.94, 3.09] | 80 | < 0.001 | 0.972 | 6467 | < 0.001 | 187 |
| TCT vs. L | 3.38 ± 0.27 [2.83, 3.92] | 13 | < 0.001 | 1.33 ± 0.12 [1.09, 1.56] | 11.4 | < 0.001 | 0.766 | 131 | < 0.001 | 40 |
| $A_{adh}$ vs L | 5.74 ± 0.81 [4.08, 7.40] | 7.1 | < 0.001 | 2.49 ± 0.36 [1.75, 3.23] | 6.9 | < 0.001 | 0.620 | 47 | < 0.001 | 29 |
| $L_S$ vs L | -0.23 ± 0.21 [-0.65, 0.20] | -1.1 | 0.29 | 0.52 ± 0.09 [0.33, 0.70] | 5.6 | < 0.001 | 0.357 | 31 | < 0.001 | 56 |
| $L_L$ vs L | -0.39 ± 0.30 [-0.99, 0.21] | -1.3 | 0.19 | 0.72 ± 0.13 [0.46, 0.98] | 5.5 | < 0.001 | 0.363 | 31 | < 0.001 | 54 |

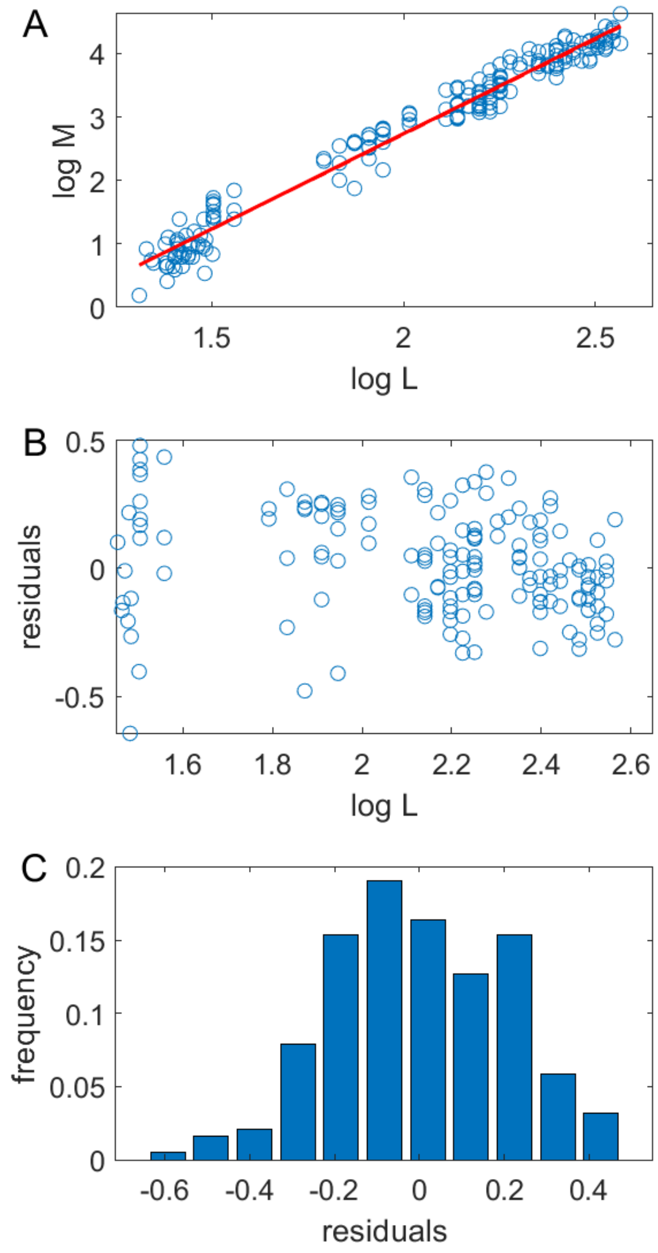


**S3 Fig. Power law fit results for spotted lanternfly nymph body mass vs length for 2021.** (A) Spotted lanternfly nymph log body mass vs log body length data (blue circles) with the best fit power law (solid red line) and isometric power law (dashed red line). (B) Fit residuals vs log L. (C) Histogram of fit residuals.

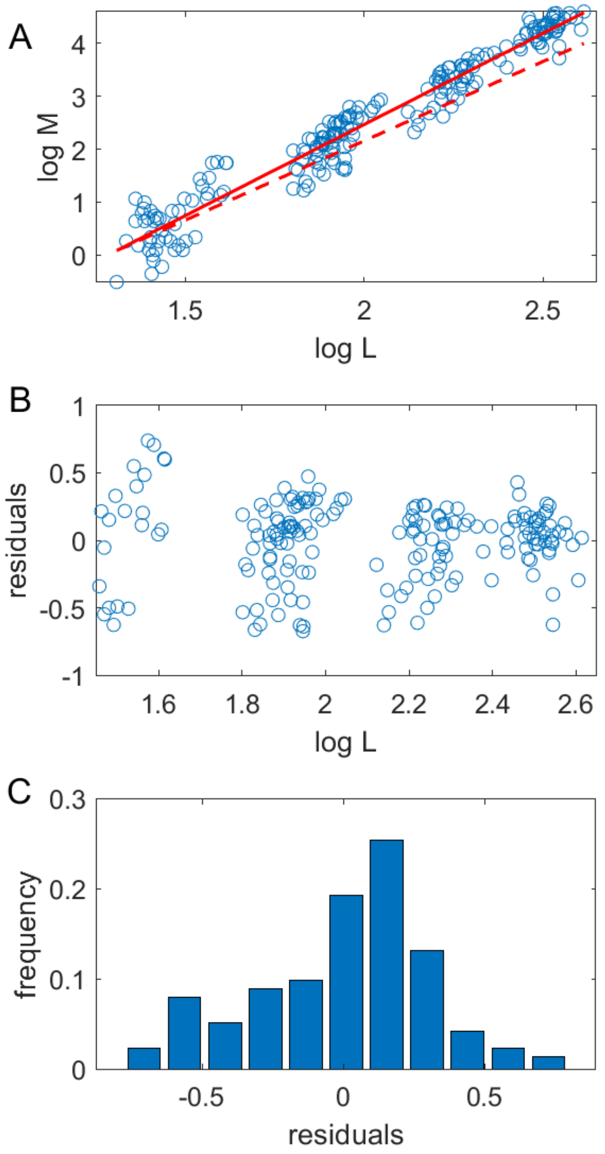


**S4 Fig. Power law fit results for spotted lanternfly nymph body mass vs length for 2022.** (A) Spotted lanternfly nymph log body mass vs log body length data (blue circles) with the best fit power law (solid red line) and isometric power law (dashed red line). (B) Fit residuals vs log L. (C) Histogram of fit residuals.
